# Supplementary material for: A Repurposed Drug Selection Pipeline to Identify CNS-Penetrant Drug Candidates for Glioblastoma
Source: Pharmaceuticals (Basel). 2024 Dec 14;17(12):1687. doi: 10.3390/ph17121687 (PMC11678797; doi:10.3390/ph17121687)
Supplement: Supplementary file 1 [file pharmaceuticals-17-01687-s001.zip › Ntafoulis et al. Supplemental figures.pdf]

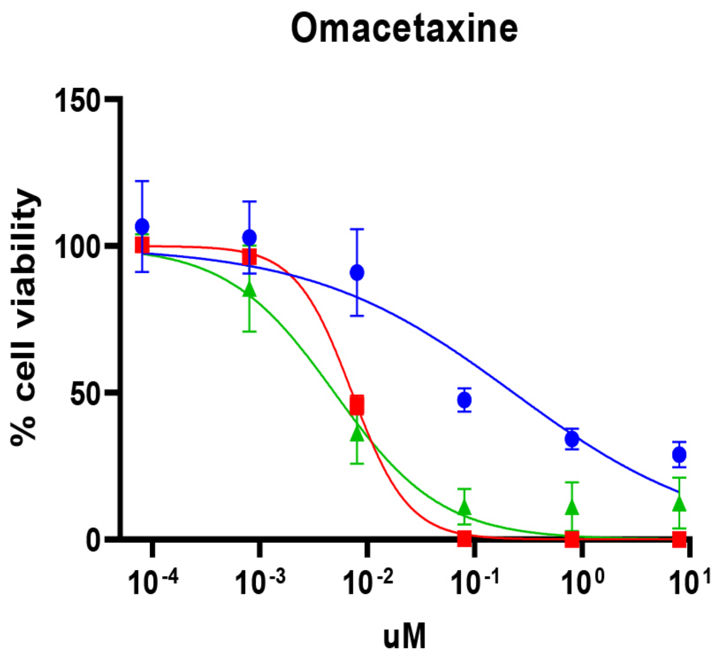

Figure S1: Dose response curves of omacetaxine mepessucinate (OMA). Dose response curves of OMA on three patient-derived GBM cultures showing percentage viability of controls (y axis) and applied OMA dose in uM (x axis).

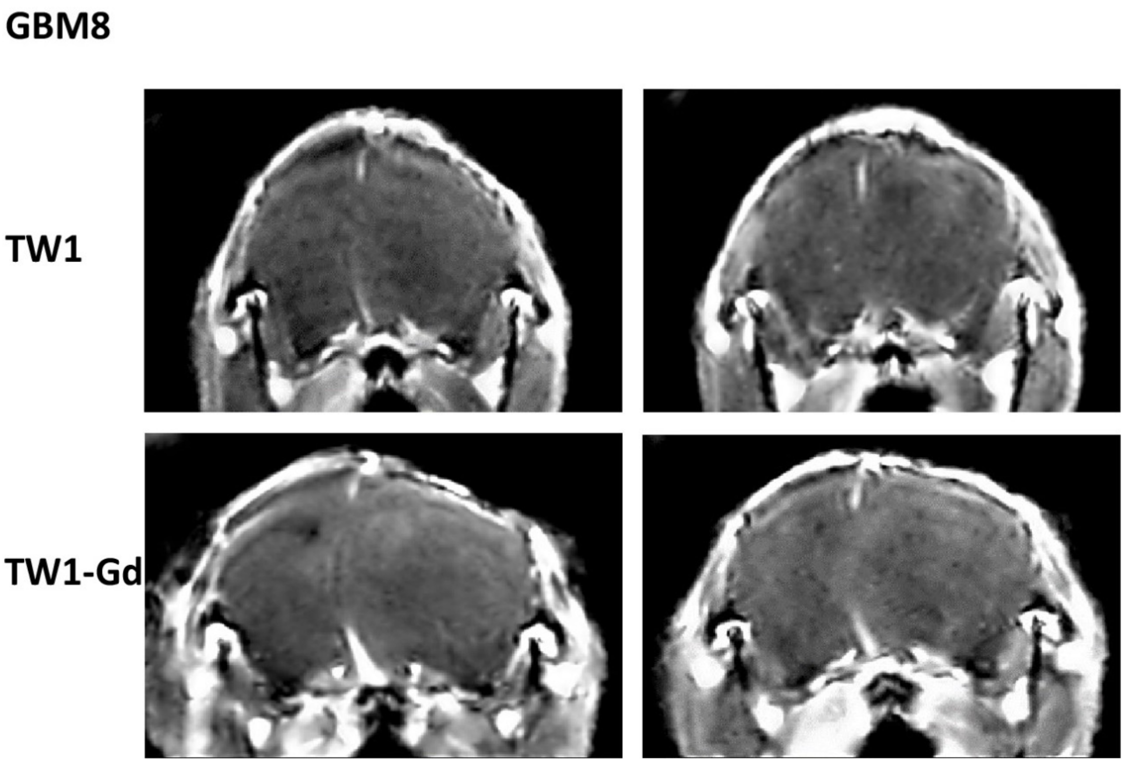

Figure S2: MRI scans of mouse brain. A coronal view of TW1 and TW1-Gd scans of a mouse brain bearing a GBM8 tumor.

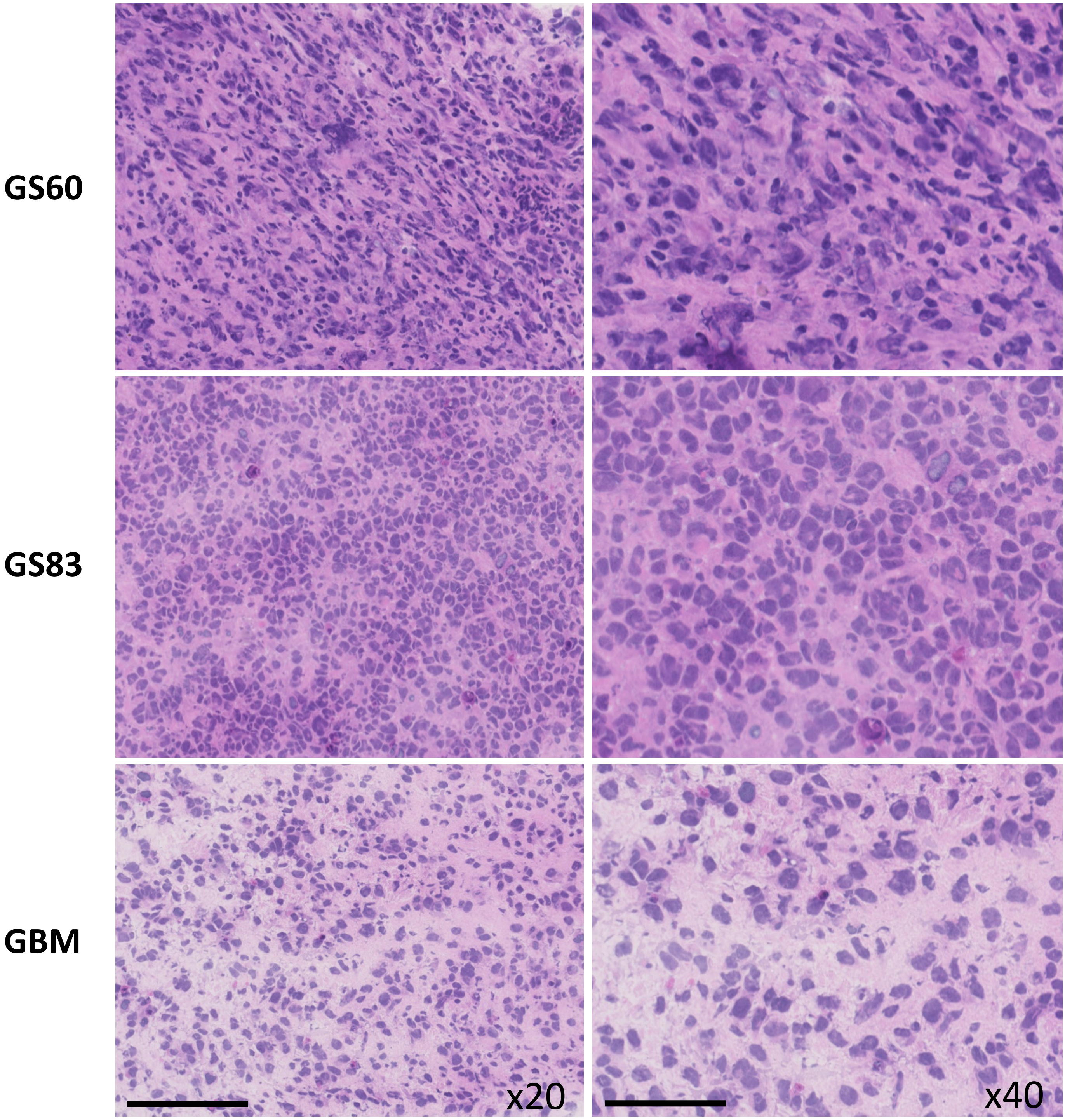

Figure S3: HE-stained sections. Hematoxylin/eosin stained sections of mouse brains bearing GS607, GS832 and GBM8 tumor. On the left, x20 magnification and scale bar 50μm, on the right x40 magnification and scale bar 100 μm.
