# Supplementary material for: A Repurposed Drug Selection Pipeline to Identify CNS-Penetrant Drug Candidates for Glioblastoma
Source: Pharmaceuticals (Basel). 2024 Dec 14;17(12):1687. doi: 10.3390/ph17121687 (PMC11678797; doi:10.3390/ph17121687)
Supplement: Supplementary file 1 [file pharmaceuticals-17-01687-s001.zip › Ntafoulis et al. Supplemental Table S1.pdf]

Table S1: Overview of drug candidates' characteristics

Drug	CNS MPO	C_{max} (uM)	Plasma protein binding (%)	P-gp substrate	BCRP substrate
Afatinib	3,27	0,05	95,00	positive	positive
Allopurinol	5,50	14,30	negligibly	negative	NA
Altretamine	6,30	3,76	NA	negative	NA
Aminolevulinic_acid_hydrochloride	5,50	129,00	12,50	negative	NA
Anastrozole	5,50	0,04	40,00	negative	NA
Arsenic_trioxide	5,00	0,91	95,00	negative	NA
Axitinib	3,17	0,16	99,00	positive	NA
Azacitidine	4,00	3,07	1,00	negative	NA
Bendamustine_hydrochloride	5,46	16,30	95,00	positive	NA
Bleomycin_sulfate	2,00	706,00	1,00	positive	NA
Bortezomib	2,58	0,31	83,00	positive	negative
Bosutinib	4,30	0,38	96,00	positive	negative
Busulfan	5,00	4,96	32,00	negative	NA
Cabazitaxel	2,75	0,27	82,00	positive	NA
Cabozantinib	2,05	4,61	99,70	positive	NA
Capecitabine	3,96	21,10	35,00	positive	NA
Carboplatin	3,65	135,00	40,00	negative	positive
Carfilzomib	1,70	5,88	97,00	positive	NA
Carmustine	5,75	19,40	80,00	negative	NA
Celecoxib	5,55	1,85	97,00	negative	NA
Chlorambucil	5,73	1,62	99,00	negative	NA
Cisplatin	2,70	14,40	90,00	negative	positive
Cladribine	4,21	0,02	20,00	negative	positive
Clofarabine	4,19	0,74	47,00	negative	positive
Crizotinib	3,30	0,91	91,00	positive	NA

Cyclophosphamide	5,75	128,00	20,00	negative	NA
Cytarabine_hydrochloride	4,00	54,40	13,00	negative	NA
Dabrafenib_mesylate	3,05	4,86	99,70	negative	positive
Dacarbazine	3,58	34,40	5,00	negative	NA
Dactinomycin	2,00	0,02	5,00	positive	positive
Dasatinib	3,31	0,26	96,00	negative	positive
Daunorubicin_hydrochloride	3,00	0,31	97,00	positive	positive
Decitabine	4,00	0,32	1,00	negative	NA
Dexrazoxane	3,80	136,00	2,00	negative	NA
Docetaxel	3,75	5,47	94,00	positive	NA
Doxorubicin_hydrochloride	2,50	15,34	75,00	positive	positive
Enzalutamide	4,53	35,70	98,00	negative	NA
Epirubicin_hydrochloride	2,50	16,60	77,00	positive	NA
Erlotinib_hydrochloride	5,14	3,15	93,00	positive	positive
Estramustine_phosphate_sodium	2,25	10,00	99,00	positive	NA
Etoposide	3,25	33,40	97,00	positive	positive
Everolimus	1,50	0,06	74,00	positive	negative
Exemestane	4,30	0,03	90,00	positive	NA
Floxuridine	4,70	0,0004	NA	negative	NA
Fludarabine_phosphate	4,00	3,70	29,00	negative	NA
Fluorouracil	5,50	426,00	18,00	negative	positive
Fulvestrant	1,50	0,04	99,00	positive	NA
Gefitinib	4,77	0,36	90,00	positive	positive
Gemcitabine_hydrochloride	3,95	89,30	10,00	negative	NA
Hydroxyurea	5,25	795,00	77,50	negative	NA
Idarubicin hydrochloride	2,51	0,12	97,00	positive	NA
Ifosfamide	5,75	431,00	negligibly	negative	NA
Imatinib	4,58	7,50	95,00	positive	positive

Irinotecan_hydrochloride	3,15	5,78	49,00	positive	positive
Ixabepilone	2,98	0,50	72,00	positive	NA
Lapatinib	1,03	4,18	99,00	positive	NA
Lenalidomide	3,60	1,74	30,00	positive	NA
Letrozole	6,00	0,41	55,00	negative	NA
Lomustine	5,90	5,00	50,00	negative	NA
Mechlorethamine_hydrochloride	4,00	0,01	NA	negative	NA
Megestrol_acetate	4,78	1,96	NA	positive	NA
Melphalan_hydrochloride	4,48	15,40	75,00	negative	negative
Mercaptopurine	5,00	0,59	19,00	negative	NA
Methotrexate	3,23	1,31	50,50	positive	positive
Methoxsalen	6,00	0,62	98,00	negative	NA
Mitomycin	4,08	2,18	22,00	positive	NA
Mitotane	2,90	43,12	6,00	negative	NA
Mitoxantrone	2,78	0,72	78,00	positive	positive
Nelarabine	4,00	16,82	25,00	negative	NA
Nilotinib	1,06	0,84	NA	negative	positive
Omacetaxine_mepesuccinate	4,00	0,043	49,00	positive	NA
Oxaliplatin	2,51	4,96	90,00	positive	positive
Paclitaxel	1,50	4,27	94,00	positive	negative
Pazopanib_hydrochloride	3,67	133,00	99,00	negative	positive
Pemetrexed	3,37	306,00	81,00	positive	NA
Pentostatin	3,73	1,82	4,00	positive	NA
Pipobroman	NA	NA	NA	negative	NA
Plicamycin	2,00	0,18	1,00	positive	NA
Pomalidomide	4,66	0,27	44,00	positive	NA
Ponatinib	2,75	0,14	99,00	positive	positive
Pralatrexate	3,12	10,30	76,50	positive	NA

Procarbazine_hydrochloride	5,25	3,13	NA	negative	negative
Raloxifene	2,20	0,001	95,00	positive	positive
Regorafenib	0,80	8,08	95,00	negative	NA
Romidepsin	2,70	0,70	93,00	positive	NA
Sirolimus	1,50	0,09	92,00	positive	NA
Sorafenib	0,94	20,10	99,50	negative	positive
Streptozocin	4,00	1,44	NA	negative	NA
Sunitinib	4,40	0,06	95,00	positive	NA
Tamoxifen_citrate	2,74	0,11	98,00	positive	positive
Temozolomide	4,35	29,87	15,00	positive	positive
Temsirolimus	1,40	0,57	87,00	positive	negative
Teniposide	3,25	23,10	95,00	positive	positive
Thioguanine	5,25	0,31	NA	negative	NA
Thiotepa	5,00	7,03	15,00	positive	negative
Topotecan_hydrochloride	3,39	0,02	35,00	positive	positive
Trametinib	2,86	0,02	97,00	negative	NA
Tretinoin	3,30	1,15	95,00	negative	NA
Uracil_mustard	5,00	NA	5,00	positive	NA
Valrubicin	2,15	0,01	99,00	positive	NA
Vandetanib	4,13	2,16	90,00	positive	positive
Vemurafenib	2,06	127,00	99,00	negative	positive
Vinblastine_sulfate	1,25	0,04	97,00	positive	NA
Vincristine_sulfate	1,25	0,01	75,00	positive	positive
Vinorelbine_tartrate	1,50	0,81	85,00	positive	NA
Vismodegib	4,70	33,90	99,00	negative	NA
Vorinostat	5,60	1,20	71,00	negative	NA

The table shows the drug characteristics of each of the 107 anti-cancer drug candidates used in the selection pipeline.
