# Supplementary material for: A Repurposed Drug Selection Pipeline to Identify CNS-Penetrant Drug Candidates for Glioblastoma
Source: Pharmaceuticals (Basel). 2024 Dec 14;17(12):1687. doi: 10.3390/ph17121687 (PMC11678797; doi:10.3390/ph17121687)
Supplement: Supplementary file 1 [file pharmaceuticals-17-01687-s001.zip › Ntafoulis et al. Supplemental Table S2.pdf]

Table S2. Summary of vascular integrity and BBB permeability of the GBM-PDX models

	P-gp	BCRP	ZO-1	GLUT1	NESTIN	TW1+Gd
**GS607**	+	+	+/-	+	+/-	Tumor enhancement
**GS832**	+	+	+	+	+	No tumor enhancement
**GBM8**	+	+	+/-	+	+	No tumor enhancement

presence (+), partial presence (+/-) or absence (-)

The table displays the presence (+), partial presence (+/-) or absence (-) of each marker used to assess the vascular integrity and blood–brain barrier (BBB) permeability in the GS607, GS832 and GBM8 patient-derived xenograft (PDX) models. Additionally, it summarizes the outcomes of the TW1 post-gadolinium MRI scans in these PDX models.
