# Supplementary material for: A Repurposed Drug Selection Pipeline to Identify CNS-Penetrant Drug Candidates for Glioblastoma
Source: Pharmaceuticals (Basel). 2024 Dec 14;17(12):1687. doi: 10.3390/ph17121687 (PMC11678797; doi:10.3390/ph17121687)
Supplement: Supplementary file 1 [file pharmaceuticals-17-01687-s001.zip › Ntafoulis et al. Supplemental Table S3.pdf]

Table S3: Comparison of omacetaxine's concentration in brain tumors with the in vitro IC50 values.

GSC	IC_{50} (nM)	[Brain tumor] (nM)	Timepoints (minutes)
**GS832**	4.9	33.0	60
		38.1	120
		21.3	240
**GS607**	22.9	37.9	60
**GBM8**	7.2	82.5	60

Table showing a comparison of omacetaxine's in vitro IC50 values alongside the corresponding concentrations of omacetaxine found in the brain tumor tissue of GS832, GS607 and GBM8 PDX models at specified timepoints.
